# IL-15 Deficient Tax Mice Reveal a Role for IL-1α in Tumor Immunity

**DOI:** 10.1371/journal.pone.0085028

**Published:** 2014-01-08

**Authors:** Daniel A. Rauch, John C. Harding, Lee Ratner

**Affiliations:** Department of Medicine, Division of Molecular Oncology, Washington University School of Medicine, Saint Louis, Missouri, United States of America; National Institute of Health - National Cancer Institute, United States of America

## Abstract

IL-15 is recognized as a promising candidate for tumor immunotherapy and has been described as both a promoter of cancer and a promoter of anti-cancer immunity. IL-15 was discovered in cells transformed by HTLV-1, the etiologic agent of adult T cell leukemia/lymphoma (ATL) and the human retrovirus that carries the Tax oncogene. We have developed the TAX-LUC mouse model of ATL in which Tax expression drives both malignant transformation and luciferase expression, enabling non-invasive imaging of tumorigenesis in real time. To identify the role of IL-15 in spontaneous development of lymphoma in vivo, an IL-15^−/−^ TAX-LUC strain was developed and examined. The absence of IL-15 resulted in aggressive tumor growth and accelerated mortality and demonstrated that IL-15 was not required for Tax-mediated lymphoma but was essential for anti-tumor immunity. Further analysis revealed a unique transcriptional profile in tumor cells that arise in the absence of IL-15 that included a significant increase in the expression of IL-1α and IL-1α-regulated cytokines. Moreover, anti-IL-1α antibodies and an IL-1 receptor antagonist (Anakinra) were used to interrogate the potential of IL-1α targeted therapies in this model. Taken together, these findings identify IL-15 and IL-1α as therapeutic targets in lymphoma.

## Introduction

### The Dual Role of IL-15 in Hematopoietic Malignancies

IL-15 is a central cytokine in lymphocyte development, hematopoietic malignancies, and immunotherapy, where it has paradoxically been described as both a promoter of cancer and a promoter of anti-cancer immunity. [1], [2] IL-15 belongs to the family of four-helix-bundle cytokines (also including IL-2, IL-4, IL-7, IL-9, and IL-21) which use receptors that share a common gamma-c chain and have unique alpha chains. While there is partial redundancy among this family of cytokines, IL-15 has emerged as one particularly suited for antitumor activity. IL-15 is an important factor in the development, homeostasis, proliferation, and activity of CD8^+^ T cells, NK cells, NKT cells, and intraepithelial T cells. [3] IL-15 also activates monocytes, macrophages, and dendritic cells; inhibits apoptosis in granulocytes and lymphocytes; promotes a persistent immune response without inducing T_reg_ activity; and represents a prime candidate for facilitating innate and durable adaptive tumor immunity. [2].

Although IL-15 is regarded as an excellent candidate for tumor therapy, it has also been characterized as a promoter of cancer. Co-discovered in HuT-102 cells transformed by HTLV-1 [4], [5], subsequent studies have confirmed the importance of IL-15 in a variety of hematopoietic malignancies and solid tumors. The potential mechanisms by which IL-15 mediates its pro-tumor activity include protecting tumor cells from apoptosis, and promoting proliferation, migration, invasion and metastasis. [6], [7] IL-15 is an important mediator of growth, and survival of the malignant cells in hematopoietic malignancies and solid tumors. [6], [8], [9] In fact, overexpression of IL-15 in transgenic mice is sufficient to cause CD8 leukemia and T-LGL or NKT leukemia. [10], [11] While IL-15 over-expression promotes leukemia/lymphoma, it is less well understood if IL-15 is a necessary prerequisite for cancer development. Similarly, IL-15 has the potential to be a high-value therapeutic target. What is less clear is whether systemic modulation of IL-15 activity represses or stimulates hematopoietic malignancies in vivo.

### TAX-LUC Mice as a Model of Human Lymphoma

HTLV-1, the etiologic agent of adult T cell leukemia/lymphoma (ATL), is a human retrovirus that carries the Tax oncogene. Tax activates viral transcription through the 5′ long terminal repeat (LTR) but is also capable of constitutively activating the NFκB pathway in infected cells which results in overexpression of IL-15. [5], [12] As a model of ATL, TAX transgenic mice, in which Tax expression is governed by the human granzyme B promoter, develop large granular lymphocytic lymphoma. [13] The TAX-LUC strain is a second generation model developed to take advantage of Tax as a strong activator of viral transcription through the HTLV-1 LTR. [14] A transgene in which firefly luciferase is driven by the HTLV-1 LTR (LTR-LUC) was introduced to make double transgenic TAX-LUC mice. Therefore, in TAX-LUC mice, Tax expression drives both tumorigenesis and luciferase expression, which can be detected non-invasively using bioluminescence imaging. Lymphoma in this model presents as subcutaneous tumors, massive splenomegaly, involvement of the bone marrow resulting in hypercalcemia and osteolytic bone lesions, and a chronic inflammatory response involving the persistent activation and recruitment of neutrophils to the tumor. [14], [15] Within the tumors, the malignant lymphoma cells comprise only 10–15% total tumor cells. The majority of the tumor is populated by infiltrating immune cells, primarily neutrophils, but also T cells, NK cells, and macrophages. Like ATL patient samples and cell lines, IL-15 mRNA is expressed in tumors that arise in TAX-LUC mice along with other NFκB inducible genes. [16] While IL-15 expression is associated with Tax tumor initiation and growth, the net effect of IL-15 expression in vivo has not yet been established.

### What is the Net Effect of IL-15 in Tax-lymphoma?

Using Tax-LUC mice as a model in which IL-15 is capable of promoting both cancer and cancer immunity, we sought to determine the effect of IL-15 loss on tumor growth in vivo. To this end an IL-15^−/−^ TAX-LUC strain was developed and examined. Based on our initial observation that IL-15 overexpression correlated with tumor growth we hypothesized that loss of IL-15 would impede tumor onset or growth. Surprisingly, IL-15 deficient TAX-LUC mice developed significantly larger tumors compared to IL-15^+/+^ TAX-LUC littermates. Further analysis of these tumors revealed effects of IL-15 loss on both the malignant population and the tumor infiltrating immune cells and led us to conclude that the net effect of IL-15 in this cancer model is promotion of tumor immunity.

## Results

### IL-15 is Not Required for Lymphomagenesis

**Figure 1:**
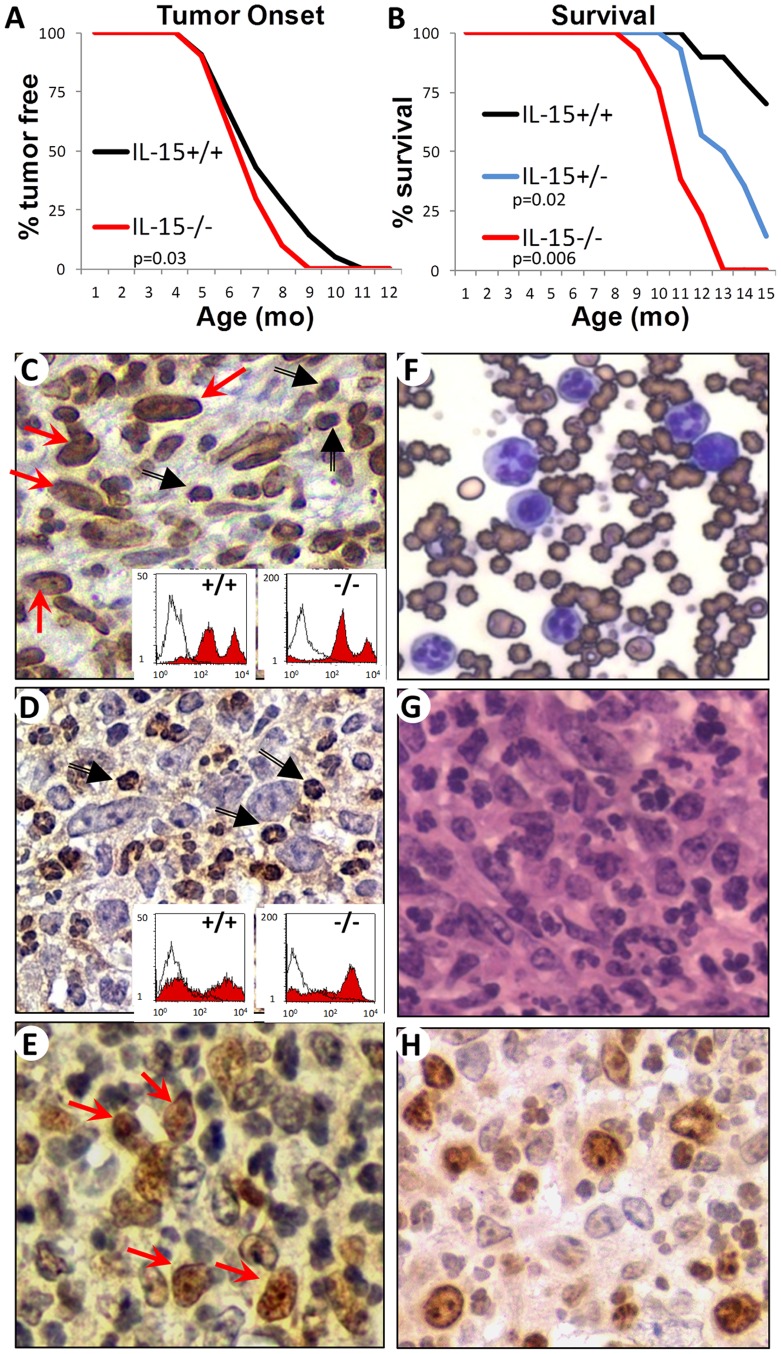
IL-15 is not required for Tax Tumorigenesis. A) Incidence of tumor onset in IL-15^−/−^ TAX-LUC mice (red line, n = 10) compared to IL-15^+/+^ TAX-LUC littermate control mice (black line, n = 21), p = 0.03 (2-tailed, paired Student’s T Test). B) Survival curve comparing IL-15^−/−^ TAX-LUC mice (red line, n = 13) and IL-15^+/−^ TAX-LUC mice (blue line, n = 14) to IL-15^+/+^ TAX-LUC littermate control mice (black line, n = 10). p = 0.006 for KO vs. WT and p = 0.02 for HET vs. WT. C) Image of CD16/32 immunohistochemistry of IL-15^−/−^ TAX-LUC tumor sections in which the malignant large granular lymphocytes (red arrows) are stained along with an admixture of tumor infiltrating neutrophils (black arrows). Insets are FACS histograms of homogenates from IL-15^+/+^ TAX-LUC or IL-15^−/−^ TAX-LUC tail tumors unstained (white curves) or stained with anti-CD16/32 FITC (red curves). D) Image of Ly6G immunohistochemistry of IL-15^−/−^ TAX-LUC tumor sections in which tumor infiltrating neutrophils (black arrows) are stained. Insets are FACS histograms of homogenates from IL-15^+/+^ TAX-LUC or IL-15^−/−^ TAX-LUC tail tumors unstained (white curves) or stained with anti-Ly6G-PE (red curves). E) Image of phosho-RelA immunohistochemistry of IL-15^−/−^ TAX-LUC tumor sections in which many of the malignant large granular lymphocytes (red arrows) show nuclear localization as a marker of NFκB activation. F) Image of a peripheral blood smear (Wright’s stain). G) Hematoxylin and eosin stained IL-15^−/−^ TAX-LUC tumor section. H) Image of Ki67 immunohistochemistry of IL-15^−/−^ TAX-LUC tumor sections.

The Tax oncogene in TAX-LUC mice is regulated by the granzyme B promoter and drives tumorigenesis and transformation of CD16^HI^ large granular lymphocytes. IL-15 expression is known to be elevated in these tumors [16] but the role of IL-15 and its effect on the tumor is unclear. To ascertain the function of IL-15 in Tax lymphoma, we created IL15^−/−^ TAX-LUC mice and observed that IL-15 is not required for tumor onset or growth. Instead, mice lacking IL-15 had a slightly accelerated rate of tumor onset (FIG. 1A) and a significantly increased mortality rate (FIG. 1B) compared to age-matched IL-15^+/+^ littermates. IL-15^+/−^ littermates had an intermediate phenotype. In the absence of IL-15, tumors were composed of CD16/32^HI^ large granular lymphocytes and CD16^LO^ neutrophils (FIG. 1C,G,H), and mice developed splenomegaly and elevated lymphocyte counts in the peripheral blood (FIG. 1F) as is typically seen in TAX-LUC mice. [14] The most distinctive aspects of Tax-tumors were conserved even in the absence of IL-15 including splenomegaly, osteolytic bone lesions, a predominant admixture of tumor infiltrating Ly6G^+^ neutrophils (FIG. 1D), and constitutive NFκB activity within the malignant LGLs (FIG. 1E). These data demonstrate that IL-15 has a dramatic effect on survival but is not required for Tax-induced tumorigenesis such that neither the type of malignancy, nor the distinctive characteristics of the tumor model were modified by the absence of IL-15.

### IL-15 Represses Tumor Growth

**Figure 2:**
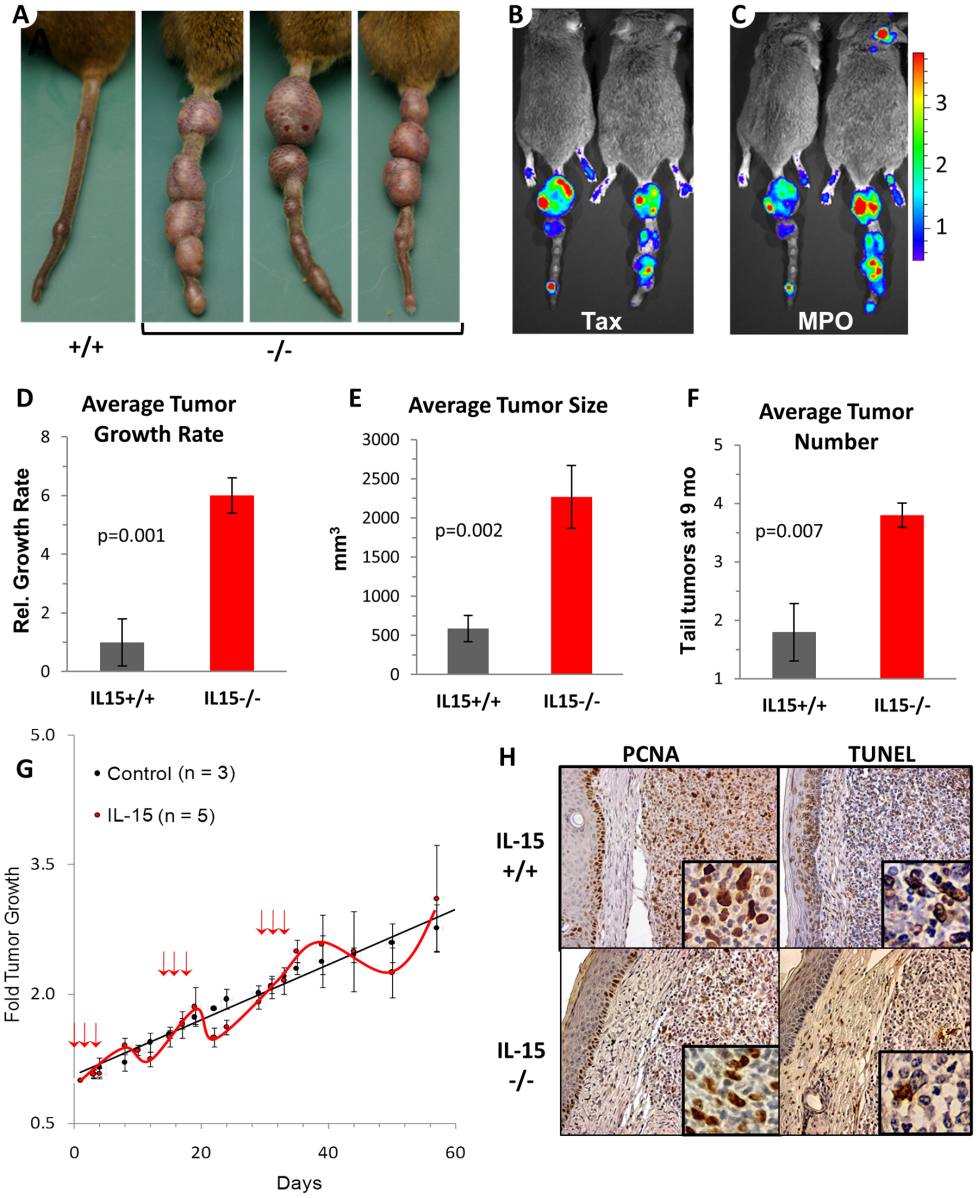
IL-15 represses tumor growth. A) Photograph of tail tumors on an IL-15^+/+^ TAX-LUC mouse and three IL-15^−/−^ TAX-LUC littermates at 8 mo of age. B,C) Bioluminescent imaging of IL-15^−/−^ TAX-LUC mice to detect Tax activity (using D-luciferin to measure LTR-Luciferase activity), and neutrophil infiltration (using Luminol to measure myeloperoxidase released from degranulating, tumor-infiltrating neutrophils). D–F) Bar graphs representing the average tumor growth rate, the average tumor size, and the average number of tail tumors per mouse on IL-15^+/+^ (n = 5), IL-15^−/−^ (n = 10) TAX-LUC mice. Weekly, bi-directional caliper measurements were taken for each tumor from tumor onset to death. Tumor volume doubling time was calculated for each tumor. To determine the growth rate, the average doubling time for IL-15^+/+^ TAX-LUC mice was set to 1 to obtain the relative average doubling time of IL-15^−/−^ TAX-LUC mice. Tumor size is shown as tumor volume in mm^3^. Number of tail tumors was calculated at 9 months of age. Error bars indicate the standard deviation and p values show 2-tailed, paired Student’s T Tests. G) The effect of murine IL-15 on tumor growth. IL-15^−/−^ TAX-LUC mice received saline (black curve, n = 3) or mIL-15 (red curve, n = 5) at times indicated by red arrows. Error bars represent standard deviation of average tumor volume as measured by calipers. H) Images of representative immunohistochemistry stains for proliferation (proliferating cell nuclear antigen; PCNA) and apoptosis (TUNEL) on IL-15^+/+^ TAX-LUC and IL-15^−/−^ TAX-LUC tail tumor sections.

Instead of exposing the cytokine as a necessity for tumor development, the absence of IL-15 dramatically accelerated tumor growth (FIG. 2A). Luciferase expression resulting from Tax activity in the large tumors (FIG. 2B) correlated with the increased levels of Tax protein and RNA in IL-15^−/−^ tumors (FIG. S1). Although IL-15^−/−^ tumors were larger, no significant increase in the overall percentage of malignant cells in these tumors was observed (FIG. 1C insets). The level of Tax expression per tumor cell was increased in the absence of IL-15. Myeloperoxidase activity of tumor infiltrating leukocytes (FIG. 2C) also correlated with areas or Tax expression. IL-15-deficient mice developed larger tumors than their IL-15^+/+^ littermates and produced more tumors with a faster growth rate (FIG. 2D–F). Moreover, administration of soluble IL-15 was sufficient to transiently reduce the rate of tumor growth in IL-15^−/−^ mice (FIG. 2G). The accelerated rate of tumor growth was not due to a greater percentage of proliferating cells within the tumor (IL-15^+/+^ = 49% vs. IL-15^−/−^ = 46%) but was instead associated with a reduced percentage of tumor cells undergoing apoptosis (37% vs. 12%) (FIG. 2H, FIG. S2). Taken together these findings establish that IL-15 represses tumor growth in Tax lymphoma by promoting tumor cell death.

### IL-15 Regulates Tumor Infiltrating Cells and Tumor Associated Cytokines

**Figure 3:**
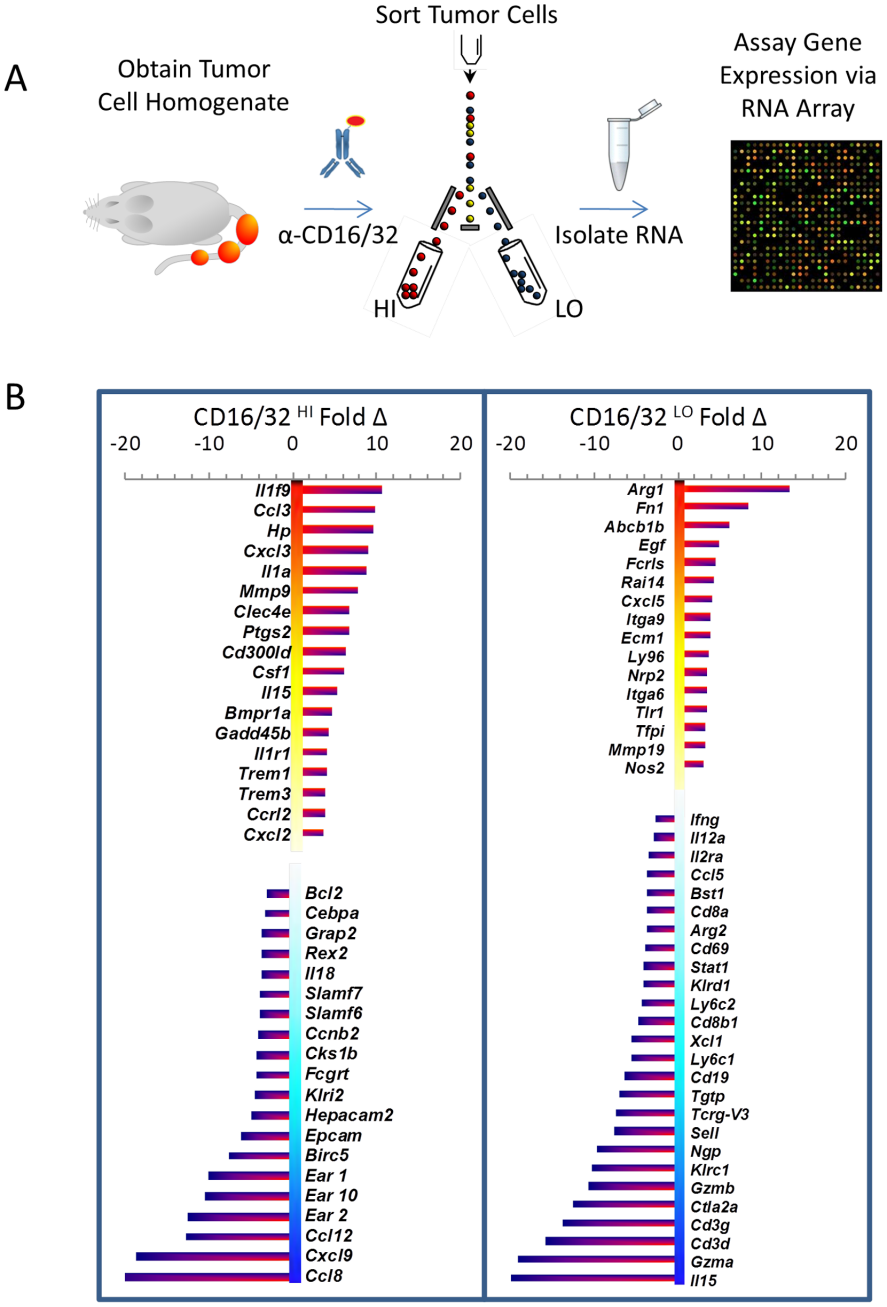
IL-15 regulates tumor infiltrating cells and tumor associated cytokines. A) Schematic representation of experimental design. RNA harvested from CD16/32^HI^ and CD16/32^LO^ cells from IL-15^+/+^ and IL-15^−/−^ TAX-LUC tail tumors were analyzed by array. B) Profiles of gene expression changes associated with the absence of IL-15 in CD16/32^HI^ and CD16/32^LO^ tumor cell populations.

To better understand the mechanism by which IL-15 regulates tumor growth we examined the effect of IL-15 loss on gene expression within two tumor cell populations, CD16/32^HI^, which is highly enriched in Tax^+^ malignant large granular lymphocytes, and CD16/32^LO^, which is a population of tumor infiltrating cells enriched in neutrophils. [17] Arrays were then performed to quantify RNA isolated from these tumor cell populations in IL-15^+/+^ and IL-15^−/−^ tumors (FIG. 3A). The average results obtained from two independent tumors of each genotype identified transcripts that were affected by the absence of IL-15 (FIG. 3B, FIG. S3). Within the malignant CD16/32^HI^ population, IL-1α and its receptor IL-1r1, IL-1α-regulated cytokines ccl3 (aka: MIP-1a) and cxcl3 (aka: MIP-2b,Gro-3), and IL-1 family member 9 were among the transcripts most highly elevated in the absence of IL-15. On the other hand, ccl8 (aka: MCP-2) and cxcl9 (aka: MIG) expression was dramatically repressed in the malignant population in the absence of IL-15.

Surprisingly, these data indicated that IL-15 gene expression was also elevated in the CD16/32^HI^ cells in IL-15^−/−^ tumors. To resolve this discrepancy, a more detailed examination was conducted of the unsummarized data from each of the 26 oligonucleotide probes complementary to the 1.25kb IL-15 mRNA on the array (FIG. S4). In IL-15^−/−^ mice, a PGK-Neo cassette replaced exons 3–5 of the 8 exons of IL-15. This region corresponds to 9 of the 26 probes on the chip. Sequence complementary to the remaining 17 probes is still present in IL-15^−/−^ mice and, on average, these 17 remaining probes reveal a 5-fold increase in IL-15 message even though the mRNA does not produce IL-15 protein. The low signal associated with the 9 probes complementary to the missing exons reflects the non-specific background signal of the chip.

Among the population of CD16/32^LO^ tumor infiltrating cells, transcripts of the neutrophil granule protein arginase 1 were markedly elevated. Tumor neutrophils in TAX-LUC mice are frequently hypersegmented with basophilic cytoplasm and ring forms are also abundant with prominent nucleoli. These characteristics were also evident in the absence of IL-15^−/−^ (FIG. 1 D,E,G,H). However, platelet clumping and an abundance of immature neutrophils in the peripheral blood in IL-15^−/−^ mice was also indicative of an impact of IL-15 loss on involved bone marrow. Reduction in gene expression in the CD16/32^LO^ population, not surprisingly, correlated with the reduction in IL-15 dependent cell lineages. Transcripts found in NK and CD8^+^ cytotoxic T cells were most reduced in the absence of IL-15 (FIG. 3B). Moreover, the decrease in CD8 cells in IL-15^−/−^ tumors was confirmed by FACS (FIG. S5).

Finally, cytokine arrays were used to examine effects of IL-15 loss at the protein level in whole tumors (FIG. S6). Tumor homogenates include epithelial, stromal, endothelial and tumor infiltrating cell types in addition to those analyzed by RNA array. The most pronounced effect of IL-15 loss on tumor-associated cytokine protein was on IL-6, which was reduced an average of 7-fold in IL-15^−/−^ Tax tumors. Since this effect was not seen in the CD16/32^+^ populations, IL-15 may be important in regulation of IL-6 in T cells or other CD16/32^−^ cells in the tumor microenvironment. It is also noteworthy that, unlike IL-1β, the elevated levels of IL-1α in normal skin keratinocytes was sufficiently high in this assay to mask any quantifiable effect of IL-15 on IL-1α protein expression within the underlying tumor. Thus, proliferating in the context of significantly reduced numbers of NK and CD8 cells, the malignant population of CD16/32^HI^ LGLs within IL-15^−/−^ tumors expressed elevated IL-1α and IL-1α-regulated transcripts and recruited activated tumor-infiltrating neutrophils.

### Loss of IL-15 Exposes IL-1α as a Regulator of Tumor Growth and Potential Therapeutic Target

**Figure 4:**
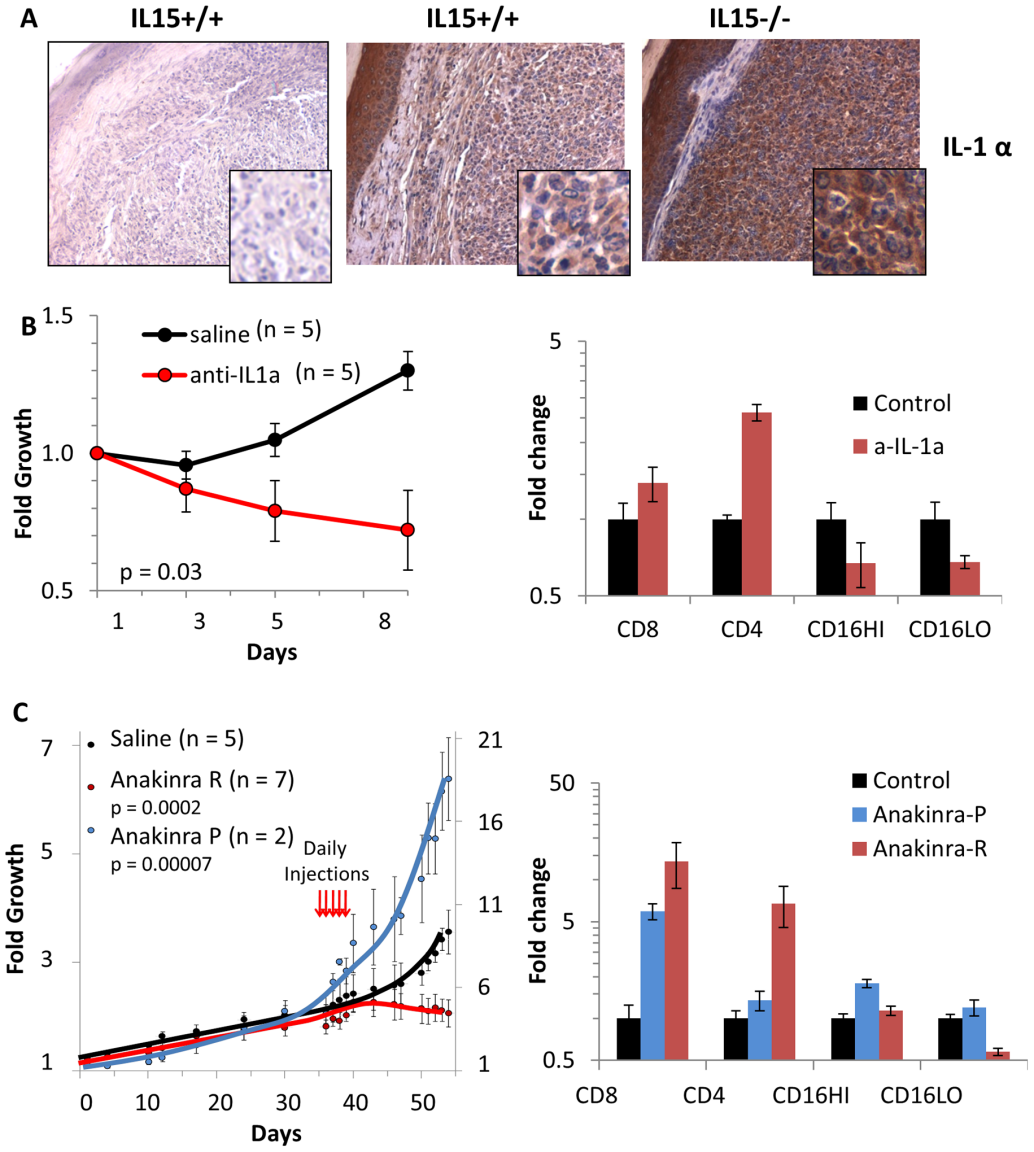
Loss of IL-15 exposes IL-1α as a regulator of tumor growth and potential therapeutic target. A) Images of IL-1α IHC in IL-15^+/+^ (middle) and IL-15^−/−^ (right) TAX-LUC tumor sections compared to control lacking primary IL-1α antibody (left). B) The effect of anti-IL-1α antibodies (red curve, n = 5) on tumor growth compared to saline (black curve, n = 5) in IL-15^+/+^ TAX-LUC mice over the course of 1 week. Error bars represent standard deviation of tumor growth, and p values represent 2-tailed, paired Student’s T Tests. The bar graph (right) represents FACS data obtained from anti-IL-1a treated tumors (red bars) or control tumors (black bars) at necropsy showing the abundance of CD8^+^, CD4^+^, CD16/32^HI^, and CD16/32^LO^ cells present in the tumor at the experimental endpoint. Y axis is log scale and error bars represent standard deviation. C) The effect of Anakinra on tumor growth compared to saline. In 7 of 9 mice Anakinra resulted in repression of tumor growth (Anakinra R, red line), while in 2 of 9 mice Anakinra resulted in promotion of tumor growth (Anakinra P, blue line). Graph is double-Y to accommodate the scale differences between Anakinra R and control (left scale) versus Anakinra P (right scale). Red arrows indicate the time points of daily Anakinra injections, error bars represent standard deviations and p values represent 2-tailed, paired Student’s T Tests. The bar graph (right) represents FACS data obtained from Anakinra treated tumors or control tumors at necropsy showing the abundance of CD8^+^, CD4^+^, CD16/32^HI^, and CD16/32^LO^ cells present in the tumor at the experimental endpoint. Y axis is log scale and error bars represent standard deviation.

Elevated levels of tumor-cell associated IL-1α protein were present in formalin-fixed paraffin embedded sections of IL-15^−/−^ tumors (Fig. 4A, Fig. S2). Interestingly, IL-1α abundance in the skin surrounding the tumor was independent of IL-15. If IL-1α expression in tumor cells is elevated in the absence of IL-15-dependent cell-mediated immunity, and it contributes to tumor cell death in the context of a competent immune system, we asked if IL-1α could be used as a therapeutic target. Two approaches were used to interrogate the potential of IL-1α targeted therapies in this model. In one experiment, neutralizing anti-IL-1α antibodies were administered to IL-15^+/+^ TAX-LUC mice and over the course of treatment, tumor growth was reduced compared to vehicle (FIG. 4B). Examination of anti-IL-1α treated tumors upon necropsy revealed an increase in tumor infiltrating CD4^+^ and CD8^+^ T cells and a decrease in the populations of both malignant LGLs and neutrophils. The antibody-based therapy could be inhibiting IL-1α signaling and/or may be targeting tumor cells for antibody dependent cell cytotoxicity (ADCC). To distinguish between these possibilities, Anakinra, a recombinant form of the IL-1 receptor antagonist, was administered to tumor bearing mice to block IL-1 signaling (FIG. 4C). In this experiment, two distinct responses were observed. Anakinra was tumoristatic in 7 of 9 tumors resulting in elevated percentages of CD4^+^ and CD8^+^ T cells and fewer tumor-associated neutrophils. However, in 2 of 9 tumors, Anakinra treatment resulted in accelerated tumor growth, higher numbers of LGLs, and had no effect on CD4^+^ T cells or neutrophils. These data identify IL-1α and IL-1 signaling as potential therapeutic targets.

## Discussion

This study was designed to determine whether IL-15 expression is a necessary prerequisite for lymphoma development in this mouse model, and whether systemic modulation of IL-15 activity is more likely to repress or stimulate similar malignancies in vivo. In the absence of IL-15, TAX-LUC mice developed larger, more aggressive tumors that resulted in accelerated mortality. The increase in tumor growth was transiently reversed in the presence of soluble IL-15 and the dearth of NK and CD8^+^ T cells in IL-15^−/−^ mice is the likely cause of this phenotype. Interestingly, rapid tumor growth immediately followed the growth inhibitory effects of soluble IL-15 leaving open the possibility that administration of IL-15 may simultaneously promote tumor cell growth and cell mediated tumor immunity in vivo.

While IL-15 was clearly not required for tumor growth in this model, IL-15 was essential for anti-tumor immunity. Because IL-15 is required for production of functional NK and CD8^+^ T cells, this model was not intended to distinguish between the effects of functional defects in NK and CD8^+^ T cells versus lack of recruitment. Instead, the tumor environment in IL-15^−/−^ mice, with reduced selective pressure of cellular cytotoxicity, revealed an unexpected transcriptional profile in the malignant cells that is apparently masked by tumor immunity. These masked targets carry potential as biomarkers capable of recruiting or activating an anti-tumor immune response. This study revealed one such target in the malignant cells to be IL-1α.

### IL-1α and Tumor Immunity

While IL-1α and IL-1β can have similar biological activities in recombinant or secreted form, these closely related cytokines seem to play very different roles in tumor immunology. [18] IL-1β is active only as a secreted product but IL-1α is also active in its cell-associated forms, as an intracellular precursor or in its membrane bound forms. Membrane-associated IL-1α can signal in a juxtracrine manner [19] and is highly immunostimulatory, [20] capable of activating CTL and NK cells and promoting anti-tumor immunity and tumor regression. [21]–[24] In the absence of cell-mediated immunity both IL-1α and IL-1β promote tumor growth. [25] However in an immune-competent host, IL-1α appears to be a key regulator of innate immunosurveillance mechanisms that promote cell-mediated repression of malignancy. [24], [26] IL-1β, on the other hand, leads to immune suppression of the host by activation and recruitment of T_reg_ cells and CD11b^+^/Gr1^+^ immature myeloid cells and it promotes systemic inflammation, tumor invasiveness and tumor angiogenesis. [21], [22], [27].

Intracellular IL-1α carries a nuclear localization signal in its N-terminus and is a chromatin associated cytokine [28] that can regulate gene expression by binding the p300-PCAF complex and activating genes in the NFκB and AP-1 pathways. [29], [30] Intracellular IL-1α can also be released during cell necrosis, where its receptor-interacting C-terminus is capable of recruiting infiltrating myeloid cells. [28] Under hypoxic conditions, HIF-1α regulates IL-1-dependent recruitment of myeloid cells [31] such that IL-1α expression correlates with the infiltration of neutrophils, whereas IL-1β is associated with the recruitment of macrophages. [28], [32] In sum, IL-1α and IL-1β are unique therapeutic targets and cell-associated IL-1α is well-suited to serve as a tumor biomarker, CTL adjuvant, and target for antibody or complement-mediated cellular cytotoxicity.

### IL-1α and HTLV Tax

Two striking characteristics of ATLL are the long period of latency that usually precedes disease onset and the low penetrance of ATL among infected carriers. Among the mechanisms thought to underlie these characteristics, one major factor is the role of CTL and host immunity. [33], [34] It has been established that the Tax oncogene is an antigenic target of the adaptive immune response in HTLV-1 infected individuals. [35] Tax is also known to activate the IL-1α promoter, [36] several mouse models have demonstrated elevated levels of IL-1α in response to Tax expression, [13], [17], [37] and HTLV-1+ T cell clones produce high levels of IL-1α in culture. [38] Interestingly, Tax expression is largely restricted or absent in primary ATL cells [39], [40] and the same is true for IL-1α. [41] Compared to IL-1β, IL-1α is also less abundant in primary tissue samples of other human malignancies including melanoma, colon carcinoma, and non-small cell lung carcinoma. [42] These data are consistent with the hypothesis that there is a selection against IL-1α expression in ATL cells, and other malignancies, in vivo. Our data provides a mechanistic explanation for these observations in ATL. Tax activity leads to IL-1α expression which recruits and activates cell-mediated killing of IL-1α -expressing, HTLV-1 infected cells. This is corroborated by the fact that depletion of IL-1α in a Tax transgenic mouse model reduced Tax-induced autoimmunity. [43] What remains to be determined is whether IL-1α-directed therapies can enhance killing of HTLV infected cells in vivo, reduce viral load, or target Tax-dependent mechanisms of ATL progression.

### Translational Implications of IL-15 and IL-1α

**Figure 5:**
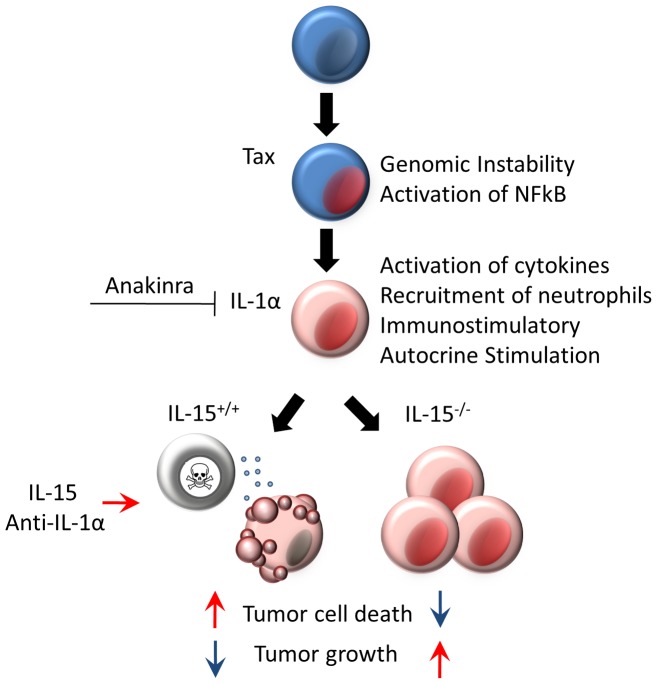
Model. A schematic representation of the principle conclusions; IL-15 is not required for tumor growth but is required for tumor immunity, IL-1α is more abundant and tumor growth is accelerated in the absence of IL-15, and therapies that target IL-1 and/or promote cell-mediated tumor immunity should be explored in lymphoma or other malignancies.

IL-15 is already recognized as a promising candidate for tumor immunotherapy. [1], [2] The principle findings in this study are that IL-15 is critical in the inhibition of tumor growth in this model and that IL-1α expression is targeted by IL-15-dependent anti-tumor activity (FIG. 5). Taken together these findings provide a rationale for testing IL-15, an anti-IL-1α antibody or a combination of both in lymphoma. The combination of IL-15 and cetuximab increased ADCC activity against triple negative breast cancer cell lines and enhanced cetuximab efficacy against HER1-positive head and neck cancer. [44], [45] IL-15 has also been shown to enhance rituximab-dependent cytotoxicity against chronic lymphocytic leukemia cells. [46] Moreover, the combination of IL-15 and anti-CD40 is more efficacious than either therapy alone in mouse models of metastatic colon cancer or metastatic renal cell carcinoma. [47]–[49] An anti- IL-1α monoclonal antibody (MABp1, Xbiotech) is currently undergoing clinical trials for treatment of advanced leukemia and other cancers, and has shown evidence of anti-tumor activity. [50] Alone and in combination, IL-15 and anti-IL-1α therapies are worth exploring against hematopoietic malignancies.

## Materials and Methods

### Animals

IL-15^−/−^ mice on a C57BL/6 background (3, Taconic) were intercrossed with TAX-LUC transgenic mice on a C57BL/6×FVB background. [14] The use of murine models and tissues in this study was carried out in strict accordance with the recommendations in the Guide for the Care and Use of Laboratory Animals of the National Institutes of Health. Mice were housed under pathogen-free conditions according to the guidelines of the Division of Comparative Medicine and all experiments were approved by the Animal Studies Committee, Washington University School of Medicine under ASC protocol #20100026.

### Histology and Immunohistochemistry

Tumor or organ tissue was fixed in 10% buffered formalin and paraffin-embedded sections were processed for immunohistochemical stains. Slides were heated to 55^o^C for 10 minutes, deparaffinized in xylene, and rehydrated in deionized water. For antigen retrieval/unmasking, slides were boiled (under pressure) in citric acid buffer (pH 6) for 15 minutes and allowed to cool to room temperature. In some cases, slides were also treated with proteinase K (20 ug/mL in TE, pH 8) for 2 minutes at room temperature, and washed with PBS. Endogenous peroxidase activity was blocked by incubating the slides in 0.3% hydrogen peroxide solution for 5 minutes. For Ki67 staining, slides were then blocked in normal equine serum (Jackson ImmunoResearch Laboratories Inc.) for 15 minutes and rinsed. Slides were soaked in rabbit polyclonal anti-Ki67 (Vector Laboratories) overnight at 4°C. Slides were then washed and incubated in ImmPRESS reagent anti-rabbit IgG (Vector Laboratories) for 30 minutes at room temperature, incubated in DAB peroxidase substrate, rinsed, and coverslipped. TUNEL (terminal deoxynucleotidyl transferase dUTP nick end labeling) staining was conducted according to manufacturer’s protocol (Trevigen). For CD16/32 staining additional blocking steps with avidin D solution (Vector Laboratories) and for 15 min at room temp, followed by biotin for 15 minutes at room temperature was included prior to incubation in primary biotin rat anti-mouse CD16/32 antibody (BD Pharmingen) overnight at 4°C. Rabbit polyclonal anti-PCNA (abcam), rabbit monoclonal anti-phosphoRel-A (Cell Signaling Technology Inc), rat anti-mouse Ly6G (eBioscience), and goat polyclonal anti-rat IL-1α (Santa Cruz biotechnology, Inc) primary antibodies were used. Negative controls lacking primary antibody were included for each stain. Sections were visualized with a Nikon Eclipse E400 microscope and digital images were obtained using a Magnafire camera and software (Optronics).

### Treatment Regimens

Soluble mIL-15: IL-15^−/−^ TAX-LUC mice received sub-cutaneous injections of saline or 100 ng of recombinant mouse IL-15 (MACS Miltenyi Biotech), 3 times per week every other week for 5 weeks. Bi-directional caliper measurements of tumors were taken three times per week for 2 months. Anti-IL-1α antibody: IL-15^+/+^ TAX-LUC mice received injections of 50 ug LEAF-purified, anti-mouse IL-1α antibody (BioLegend) or saline i.p. every other day for one week and tumors were measured with calipers. Anakinra: IL-15^+/+^ TAX-LUC received daily i.p. injections of 30 mg Anakinra (Swedish Orphan Biovitrum AB) or saline. Tumors were measured for 35 days prior to treatment and 15 days after treatment with calipers.

### Bioluminescent Imaging

The IVIS100 system (Xenogen) was used to image bioluminescence in anesthetized mice (isoflurane inhalation). Standard imaging parameters included D-luciferin dose 50 mg i.p; luminol dose 200 mg/kg i.p; exposure 300 sec; binning 4; f/stop 1; no optical filter. Color scale unless otherwise indicated is ×10^4^ photons/sec/cm^2^/sr.

### Flow Cytometry

Cell suspensions derived from organs or tumor homogenates from IL-15^−/−^ or IL-15^+/+^ mice were blocked with mouse BD Fc Block (Rat anti-mouse CD16/32; BD Pharmingen) for 10 minutes and stained with anti-CD16/32-FITC, anti-CD4-APC, anti-CD8-PE, and anti-Ly6G-PE antibodies (eBioscience) for 30 minutes at 4°C, washed, filtered and analyzed on a BD FACSCalibur (Becton Dickinson).

### RNA Array

Tail tumors were excised from two IL-15^−/−^ TAX-LUC mice and two IL-15^+/+^ TAX-LUC mice. Single cell suspensions were made, counted, re-suspended in PBS+1%BSA, blocked with Fc block, stained with anti-CD16/32 for 30 min at 4°C, and sorted using Sony iCyt Reflection Cell Sorter. Total RNA was harvested from CD16^HI^ and CD16^LO^ tumor cell populations using RNAeasy (Qiagen), quantified by spectrophotometry, and submitted for array analysis (Affymetrix Gene Chip, Whole Transcript 1.0ST). Averaged signal intensities for each probe set were analyzed (Partek) comparing CD16/32^HI^ from each genotype and CD16/32^LO^ sets from each genotype by 1-way ANOVA and then filtered for expression changes >3-fold. Original data files are available (GEO accession # GSE46072).

### Protein Array

Tail tumors and normal tail tissue from IL-15^−/−^ and IL-15^+/+^ TAX-LUC mice were excised and homogenized in PBS with protease inhibitors (10 µg/mL Aprotinin, 10 µg/mL Leupeptin, and 10 µg/mL Pepstatin) and Triton X-100 was added to a final concentration of 1%. Samples were then frozen at −80°C, thawed, centrifuged at 10,000×g for 5 minutes to remove cellular debris, quantified, and analyzed using a Proteome Profiler Mouse Cytokine Antibody Array (R&D Systems) according to manufacturer’s instructions.

### RT-PCR

2 µg of RNA obtained from sorted cells was subjected to DNase I digestion (Invitrogen) and RT-PCR was carried out using SuperScript III First-Strand Synthesis system (Invitrogen) according to manufacturer’s instructions. 100 ng of cDNA template was used for each reaction. Qualitative RT-PCR was performed using a two-phase, step-down PCR method; 16 cycles of 94°C for 30 s, 64°C (−0.5°C/cycle) for 30 s, 72°C for 30 s, followed by 10 cycles of 94°C for 30 s, 56°C for 30 s, 72°C for 30.

### Western Blot

Tumor cells harvested from IL-15^−/−^ and IL-15^+/+^ TAX-LUC mice were centrifuged, rinsed, resuspended in RIPA buffer, sonicated, boiled in SDS buffer for 15 min, and run on 15% SDS-PAGE at 4°C. Blots were probed using a rabbit anti-Tax primary antibody.

## Supporting Information

Figure S1 **Tax Expression.** RNA and protein obtained from tumor cells harvested from IL-15^−/−^ and IL-15^+/+^ TAX-LUC mice was used to detect Tax RNA by RT-PCR and Tax protein by Western Blot normalized against GAPDH and actin loading controls respectively.
(TIF)

Figure S2 **TUNEL, PCNA, and IL-1α IHC in IL-15^+/+^ and IL-15^−/−^ tumors.** Images of representative immunohistochemistry (IHC) stains for apoptosis (Terminal deoxynucleotidyl transferase dUTP nick end labeling; TUNEL), proliferation (proliferating cell nuclear antigen; PCNA) and interleukin-1 alpha (IL-1α) on IL-15^+/+^ TAX-LUC (top row) and IL-15^−/−^ TAX-LUC (bottom row) tail tumor sections.
(TIF)

Figure S3 **RT-PCR confirmation of selected differentially expressed mRNAs.** RNA was obtained from CD16/32^HI^ and CD16/32^LO^ sorted tumor cells harvested from IL-15^−/−^ and IL-15^+/+^ TAX-LUC mice.
(TIF)

Figure S4 **IL-15 mRNA is elevated in the malignant CD16/32^HI^ cells in IL-15^−/−^ Tax tumors.** A) Raw (“unsummarized”) data from each of the 26 oligonucleotide probes complementary to the 1.25 kb IL-15 mRNA on the array. Red bars and black bars represent data obtained from CD16/32^HI^ cells from tail tumors arising in IL-15^−/−^ and IL-15^+/+^ TAX-LUC mice respectively. B) Alignment showing the locations of each of the 26 oligonucleotide probes relative to the exon locations in the mRNA. In IL-15^−/−^ mice the PGK Neo cassette replaces exons 3–5 of the IL-15 mRNA which corresponds to probes 7–15 in the array. Probes 7–15 in the IL-15^−/−^ tumors constitute the background of the array and average a 1.54 fold decrease vs. IL-15^+/+^ compared to the 5.26 fold increase in the average of the remaining probes.
(TIF)

Figure S5 **Tumor infiltrating CD8^+^ cells are reduced in IL-15^−/−^ Tax tumors.** Dot plot histograms of CD4^+^ (Y-axis) and CD8^+^ (X-axis) cells present in tumor homogenates from IL-15^+/+^ and IL-15^−/−^ Tax tumors.
(TIF)

Figure S6 **Cytokines elevated in Tax Tumors in the presence and absence of IL-15.** Lysates were obtained from normal tail tissue and whole tail tumors from IL-15^−/−^ and IL-15^+/+^ mice and analyzed using the mouse Proteome Profiler. Representative images are shown from one array next to densitometry analysis averaging the results of n = 5 IL-15^−/−^ arrays. Densitometry value in normal IL-15^−/−^ tail tissue is shown and set to 1. A 7-fold decrease in IL-6 protein was consistently detected in IL-15^−/−^ tumors compared to IL-15^+/+^ tumors.
(TIF)
