# Supplementary figures and images for: IL-15 Deficient Tax Mice Reveal a Role for IL-1α in Tumor Immunity
Source: PLoS One. 2014 Jan 8;9(1):e85028. doi: 10.1371/journal.pone.0085028 (PMC3885672; doi:10.1371/journal.pone.0085028)

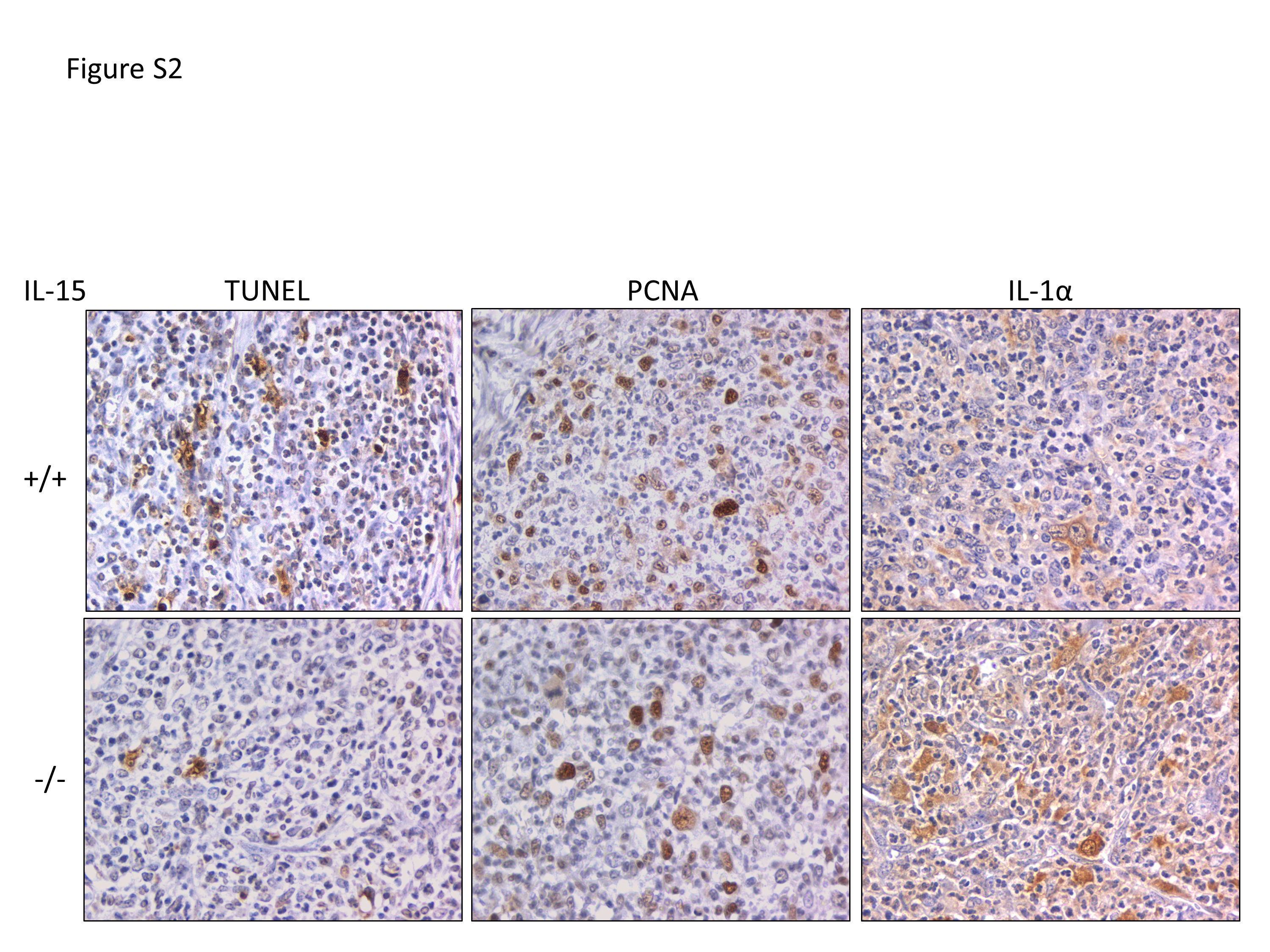

Supplement: Figure S2 — TUNEL, PCNA, and IL-1α IHC in IL-15+/+ and IL-15−/− tumors. Images of representative immunohistochemistry (IHC) stains for apoptosis (Terminal deoxynucleotidyl transferase dUTP nick end labeling; TUNEL), proliferation (proliferating cell nuclear antigen; PCNA) and interleukin-1 alpha (IL-1α) on IL-15+/+ TAX-LUC (top row) and IL-15−/− TAX-LUC (bottom row) tail tumor sections. (TIF) [file pone.0085028.s002.tif]

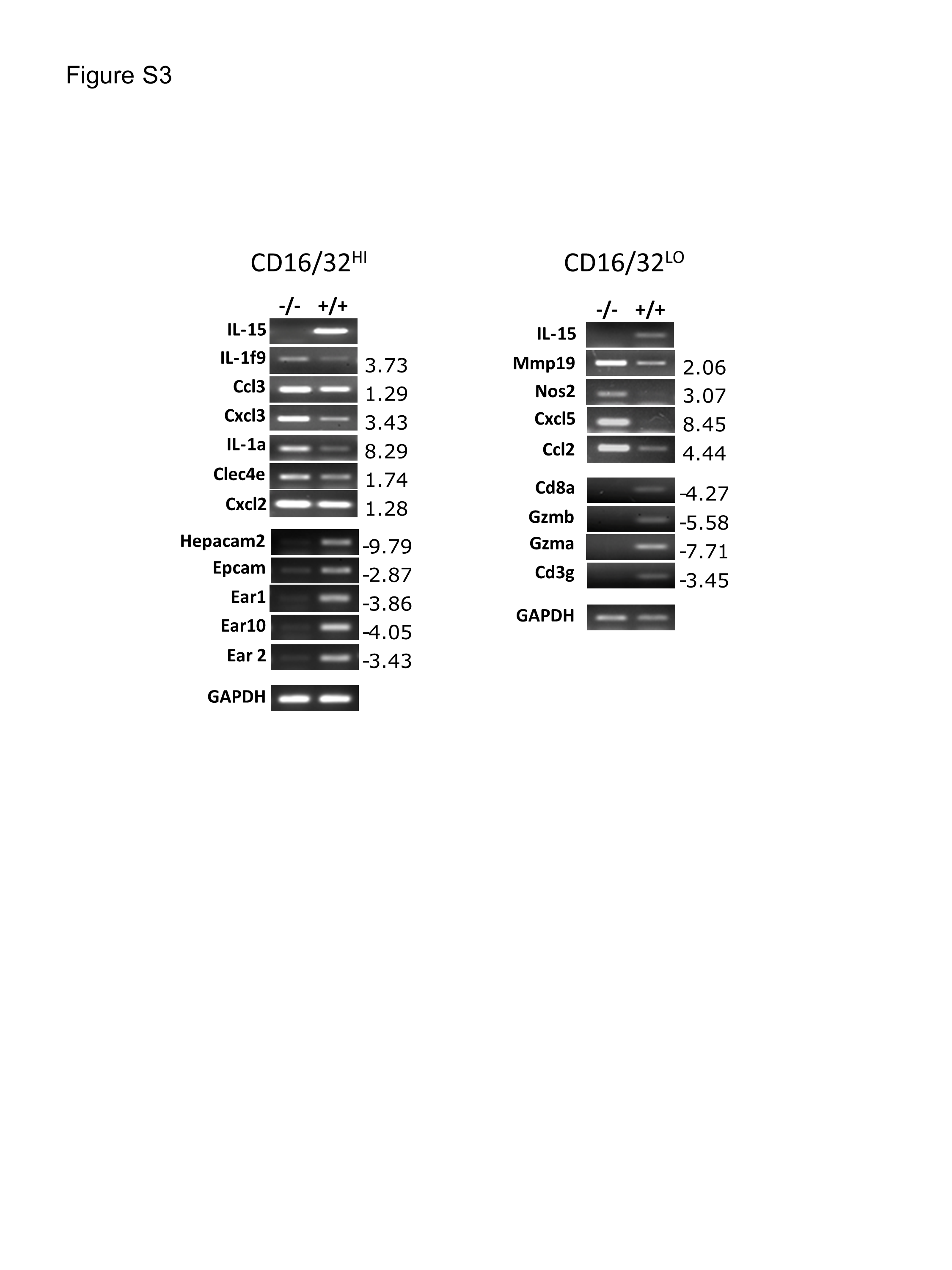

Supplement: Figure S3 — RT-PCR confirmation of selected differentially expressed mRNAs. RNA was obtained from CD16/32HI and CD16/32LO sorted tumor cells harvested from IL-15−/− and IL-15+/+ TAX-LUC mice. (TIF) [file pone.0085028.s003.tif]

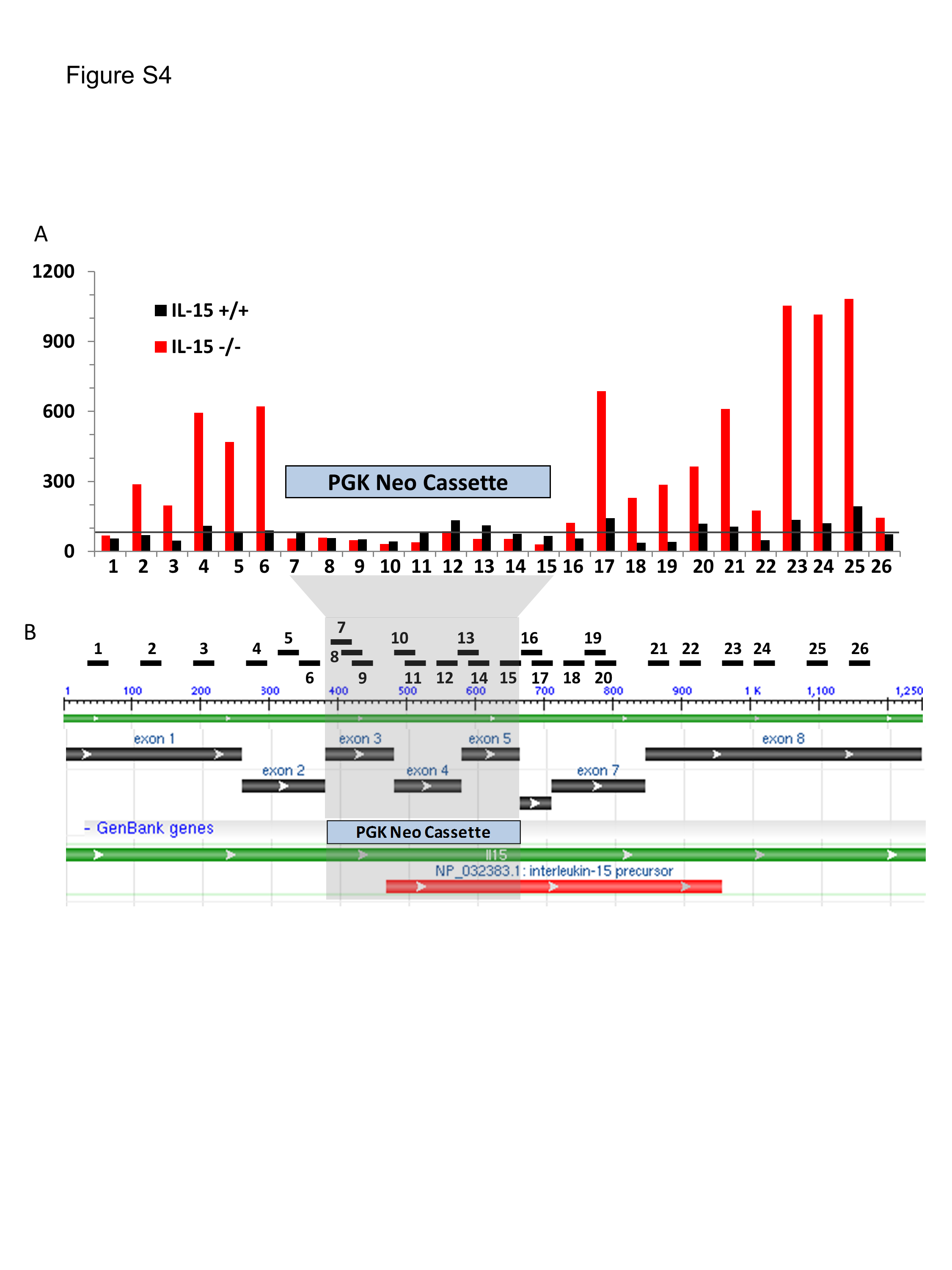

Supplement: Figure S4 — IL-15 mRNA is elevated in the malignant CD16/32HI cells in IL-15−/− Tax tumors. A) Raw (“unsummarized”) data from each of the 26 oligonucleotide probes complementary to the 1.25 kb IL-15 mRNA on the array. Red bars and black bars represent data obtained from CD16/32HI cells from tail tumors arising in IL-15−/− and IL-15+/+ TAX-LUC mice respectively. B) Alignment showing the locations of each of the 26 oligonucleotide probes relative to the exon locations in the mRNA. In IL-15−/− mice the PGK Neo cassette replaces exons 3–5 of the IL-15 mRNA which corresponds to probes 7–15 in the array. Probes 7–15 in the IL-15−/− tumors constitute the background of the array and average a 1.54 fold decrease vs. IL-15+/+ compared to the 5.26 fold increase in the average of the remaining probes. (TIF) [file pone.0085028.s004.tif]

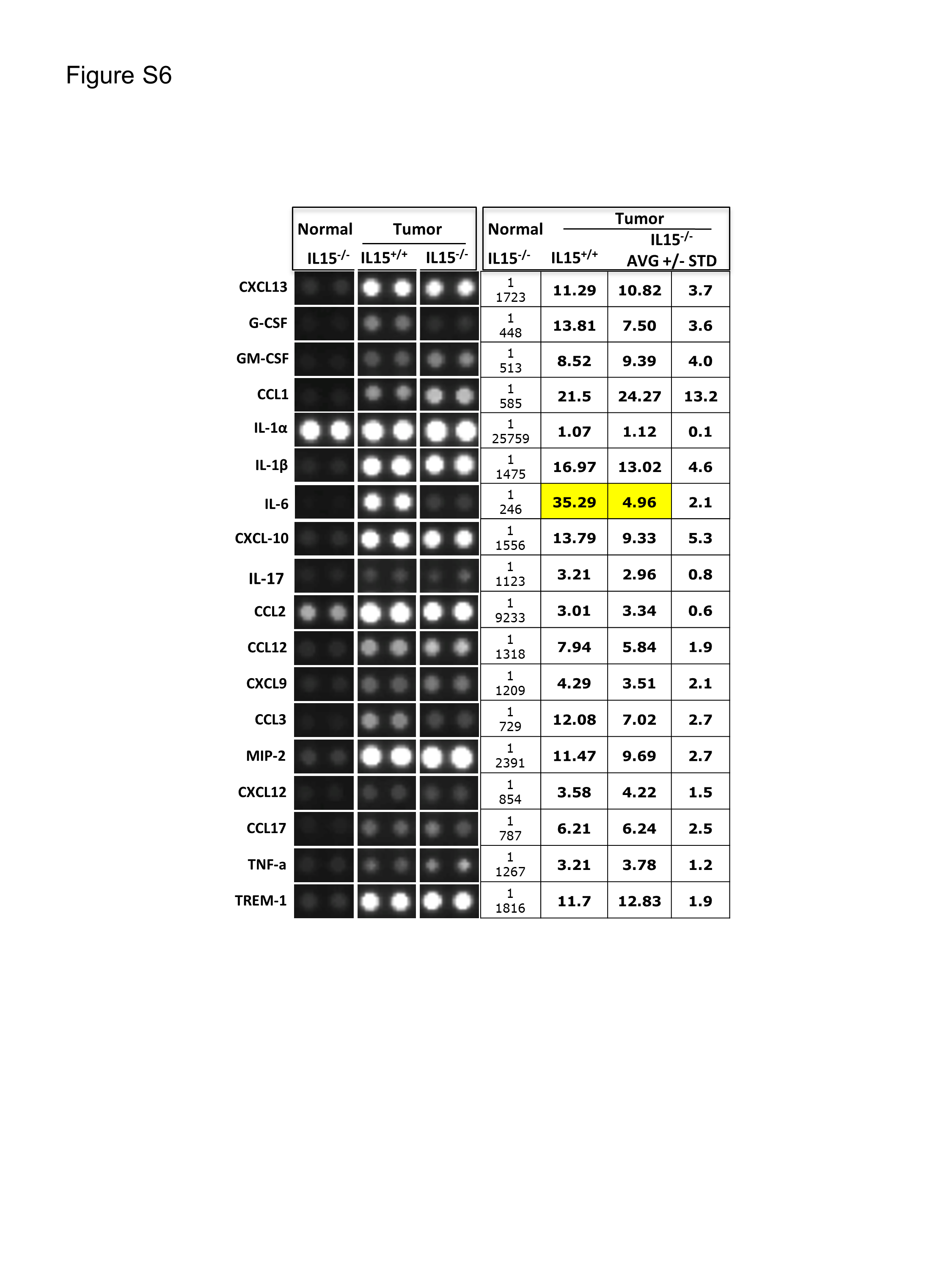

Supplement: Figure S6 — Cytokines elevated in Tax Tumors in the presence and absence of IL-15. Lysates were obtained from normal tail tissue and whole tail tumors from IL-15−/− and IL-15+/+ mice and analyzed using the mouse Proteome Profiler. Representative images are shown from one array next to densitometry analysis averaging the results of n = 5 IL-15−/− arrays. Densitometry value in normal IL-15−/− tail tissue is shown and set to 1. A 7-fold decrease in IL-6 protein was consistently detected in IL-15−/− tumors compared to IL-15+/+ tumors. (TIF) [file pone.0085028.s006.tif]
